# Serum Clusterin and Complement Factor H May Be Biomarkers Differentiate Primary Sjögren's Syndrome With and Without Neuromyelitis Optica Spectrum Disorder

**DOI:** 10.3389/fimmu.2019.02527

**Published:** 2019-10-25

**Authors:** Lin Qiao, Chuiwen Deng, Qian Wang, Wen Zhang, Yunyun Fei, Yan Xu, Yan Zhao, Yongzhe Li

**Affiliations:** ^1^Department of Internal Medicine, Peking Union Medical College Hospital, Chinese Academy of Medical Sciences & Peking Union Medical College, Beijing, China; ^2^Key Laboratory of Rheumatology and Clinical Immunology, Department of Rheumatology and Clinical Immunology, Peking Union Medical College Hospital, Chinese Academy of Medical Sciences & Peking Union Medical College, Ministry of Education, Beijing, China; ^3^Department of Neurology, Peking Union Medical College Hospital, Chinese Academy of Medical Sciences & Peking Union Medical College, Beijing, China; ^4^Department of Clinical Laboratory, Peking Union Medical College Hospital, Chinese Academy of Medical Sciences & Peking Union Medical College, Beijing, China

**Keywords:** primary Sjögren's syndrome, proteome, neuromyelitis optica spectrum disorder, clusterin, complement factor H

## Abstract

**Background:** Neuromyelitis optica spectrum disorder (NMOSD) is a neurological complication of primary Sjögren's syndrome (pSS).

**Objective:** We aimed to explore potential serological differences between pSS patients with and without NMOSD.

**Methods:** There were 4 pSS patients with NMOSD and 8 pSS patients without NMOSD enrolled as the screening group for two-dimensional difference gel electrophoresis (DIGE) analysis. Then differential expressed protein spots between groups were identified by MALDI-TOF/TOF MS. The levels of the identified potential biomarkers were verified by ELISA in a second independent cohort including 22 pSS patients with NMOSD, 26 pSS without NMOSD and 30 NMOSD patients.

**Results:** Nine proteins were identified significantly differently expressed (more than 1.5-fold, *p* < 0.05) between these two groups. Serum levels of clusterin and complement factor H (CFH) were further verified by ELISA. Results showed that the serum clusterin was significantly higher in NMOSD with pSS than without (298.33 ± 184.52 vs. 173.49 ± 63.03 ng/ml, *p* < 0.01), while the levels of CFH were lower in pSS patients with NMOSD than without (24.19 ± 1.79 vs. 25.87 ± 3.98 ng/ml, *p* < 0.01).

**Conclusion:** This is the first study of serological comparative proteomics between pSS patients with and without NMOSD. Serum clusterin and CFH might be potential biomarkers for pSS patients with NMOSD and play important role in the pathogenesis of the disease but needs further verification.

## Introduction

Primary Sjögren's syndrome (pSS) is a systemic autoimmune disease characterized by dry eyes and dry mouth. About 40% of pSS patients were found to be complicated with neuromyelitis optica spectrum disorder (NMOSD) (1). NMOSD is an inflammatory demyelinating spectrum disorder of the central nervous system characterized by severe attacks of optic neuritis and myelitis. However, the pathogenesis of pSS complicated with NMOSD has not been elucidated yet (2).

Generally, the autoimmune response appears to be more intense in the NMO group with SS (3). The prognosis of NMOSD patients complicated with autoimmune diseases was considered to be more poor than NMOSD patients without (4–6). Therefore, early diagnosis and treatment of pSS patients with NMOSD will improve their prognosis. However, the diagnosis of pSS patients with NMOSD, especially NMOSD, was confronted with problems that the specific serum biomarker, anti-aquaporin4 (AQP4) antibody was not sensitive enough (7). The similarity of sensitivity and specificity of AQP4 antibody in idiopathic NMOSD patients and NMOSD associated with autoimmune diseases is indicative of two distinct diseases with sequential or simultaneous incidences (8). Moreover, the symptoms of the central nervous system behave before mucosal symptoms and detection of anti-SSA/SSB antibody in pSS patients with NMOSD (9–11), which may result in ignoring the existence and treatment of pSS. Exploring serum biomarker of pSS with NMOSD will not only be useful for making its clinical decision but also help further illustrate the pathogenesis.

As we know, proteomics has been widely used in exploring diagnosis and prognosis biomarkers of many diseases. Previously, we have applied proteomics in establishing classification tree models and differential diagnosis biomarkers for autoimmune disease (12–15). Recently, we illustrated the pathogenesis of primary biliary cholangitis by using label-free proteomics (16). Among these researches, we found that the two-dimensional difference gel electrophoresis (DIGE) is a more sensitive and high-throughput based two-dimensional gel electrophoresis (2-DE) method (17).

In this research, we aimed to investigate serological differences between pSS patients with and without NMOSD by using DIGE, to established important biomarkers or primarily demonstrated the pathogenesis of pSS patients with NMOSD.

## Materials and Methods

### Eligibility Criteria and Study Design

Serum samples were collected at Peking Union Medical College Hospital from June 2013 to December 2013. Participants met diagnosis criteria: pSS and NMOSD were diagnosed according to the revised version of classification criteria for pSS proposed by the American-European Consensus Group (18) and the Wingerchuk criteria (1, 19), respectively, and not receive immunosuppressive treatment could be included in this study. Four pSS patients with NMOSD and eight pSS patients without NMOSD were selected as the screening group for DIGE analysis. A second independent cohort including 22 pSS patients with NMOSD, 26 pSS without NMOSD, and 30 NMOSD patients was recruited for the verification of the DIGE results (Table 1). The Ethics Committee of the Peking Union Medical College Hospital approved this study, and all methods were performed in accordance with relevant guidelines and regulations. Written informed consent was obtained from all participants.

**Table 1:** Characteristics of all recruited patients.

**Study group**	**Characteristics**	**Patients**		
		**pSS without NMOSD**	**pSS with NMOSD**	**NMOSD**
**Screening group**				
	Total number	8	4	NA
	Sex ratio (F:M)	7:1	4:0	
	Average of age disease onset (years)	47.6 ± 15.7	32.5 ± 8.4	
	Average duration (months, median)	120	10	
	Dry mouth	8 (100)	4 (100)	
	Dry eyes	8 (100)	3 (75)	
	Objective xerostomia	8 (100)	4 (100)	
	Postive ocular tests	8 (100)	3 (75)	
	Positive salivary gland biopsy	8 (100)	4 (100)	
	ANA positive	8 (100)	3 (75)	
	Anti-Ro/SSA positive	8 (100)	4 (100)	
	Anti-La/SSB positive	6 (75)	1 (25)	
**Verification group for CFH**				
	Total number	22	22	NA
	Sex ratio (F:M)	21:1	8:3	
	Average of age disease onset (years)	50 ± 10.3	40 ± 11.2	
	Average duration (months, median)	48	36	
	Dry mouth	19 (86.3)	18 (81.2)	
	Dry eyes	17 (77.3)	16 (72.3)	
	Objective xerostomia	20 (90.1)	19 (86.4)	
	Postive ocular tests	20 (90.1)	19 (86.4)	
	Positive salivary gland biopsy	14/16 (87.5)	15/18 (83.3)	
	ANA positive	22 (100)	22 (100)	
	Anti-Ro/SSA positive	22 (100)	22 (100)	
	Anti-La/SSB positive	9 (40.9)	6 (27.3)	
**Verification group for clusterin**				
	Total number	26	22	30
	Sex ratio (F:M)	11:2	8:3	23:7
	Average of age disease onset (years)	47 ± 10.3	40 ± 11.2	40 ± 14.6
	Average duration (months, median)	46	36	38
	Dry mouth	23 (88.5)	18 (81.2)	0 (0)
	Dry eyes	19 (73.1)	16 (72.3)	0 (0)
	Objective xerostomia	24 (92.3)	19 (86.4)	NA
	Postive ocular tests	23 (88.5)	19 (86.4)	NA
	Positive salivary gland biopsy	17/19 (89.5)	15/18 (83.3)	NA
	ANA positive	26 (100)	22 (100)	5 (16.7)
	Anti-Ro/SSA positive	26 (100)	22 (100)	0 (0)
	Anti-La/SSB positive	10 (38.5)	6 (27.3)	0 (0)

*pSS, primary Sjögren's syndrome; NMOSD, Neuromyelitis optica spectrum disorder; CFH, complement factor H*.

### Sample Collection and Preparation for 2-DE Analysis

Blood samples were collected from the cubital vein under fasting conditions and dispensed into 5-mL pro-coagulation tubes with gel (Becton, Dickinson and Company, UK). Blood samples were centrifuged at 1,000 × g for 5 min within 6 h at 4°C. Sera were stored at −80°C until use.

High abundant proteins from the serum samples included for DIGE analysis were removed by applying the Agilent Human 14 Multiple Affinity Removal System Column (Agilent Technologies, CA, USA) according to the manufacturer's protocol. The 14 most abundant proteins (albumin, immunoglobulin G, α1-antitrypsin, immunoglobulin A, transferrin, haptoglobin, fibrinogen, α2-macroglobulin, α1-acid glycoprotein, immunoglobulin M, apolipoprotein A-1, apolipoprotein A-II, complement C3, and transthyretin) were removed by this method. Then protein concentrations of the depleted serum samples were detected with the Bradford assay (Bio-Rad, USA).

### DIGE

Depleted serum samples from the patients of screening group were analyzed by DIGE, which was performed according to our previous research (14). Briefly, 50 μg of each sample were pooled and used as an internal standard that will run on each gel to control gel-to-gel variation. Then protein extracts from pSS without NMOSD, pSS with NMOSD, and the internal standard were labeled with DIGE fluors, Cy3, Cy5, and Cy2 (GE Healthcare, NJ, USA), respectively, according to the manufacturer's protocols (20). The Cy2, Cy3, and Cy5 labeled samples were loaded onto a 13 cm, pH 3–10 Immobiline Drystrips (GE Healthcare), with the setting of electrophoresis conditions as follow: 30 V for 12 h, 500 V for 1 h, 1,000 V for 1 h, 8,000 V for 8 h, and 500 V for 4 h. After equilibration, the strips were loaded on a 12.5% polyacrylamide gel for second separation with the setting of electrophoresis, 15 mA for 10 min, and then at 30 mA until the samples were 5 mm from the bottom of the gel. The gel was then scanned by Typhoon 9400 (GE Healthcare). DeCyder 6.5 sofeware (GE Healthcare) was used for image analysis. The dye intensity of protein spots from pSS patients with and without NMOSD were compared by Biological Variation Analysis module using a *t*-test, and *p*-values < 0.05 were considered as significant. Spots that were significantly different between groups (more than 1.5-fold) were selected and identified.

One milligram of internal standard was run on a 2-DE gel to gain all the selected spots. After destained, dehydrated and proteins digested, gel pieces of these spots were prepared for protein identification. A 4800 Plus MALDI TOF/TOF^TM^ Analyzer (AB SCIEX, USA) was used for protein identification. Bioinformation including MS/MS queries were run on the MASCOT search engine 2.2 (Matrix Science).

### ELISA Validation

ELISA was used to verify the reliability of DIGE results. In particular, we investigated the expression of clusterin and complement factor H (CFH). The validation experiments were performed using the commercially available ELISA Kit for human clusterin (Abcam, Cambridge, UK) and CFH (Abcam, Cambridge, UK). Twenty-six pSS patients without NMOSD, 22 pSS patients with NMOSD, and 30 NMOSD patients were used to determine the level of clusterin. And 22 pSS patients without NMOSD and 22 pSS patients with NMOSD were used to determining the level of CFH. For the quantification of clusterin and CFH, 10 μL of each sample was diluted 1:500 and 1:1,000 with sample diluent, respectively. Serum samples and protein standards were added to pre-designated wells on the 96-well microtiter plate. The assay was performed according to the manufacturer's specifications. The absorbance was read by a microplate reader at 450 nm. And the assay results were analyzed using the GraphPad Prism software and SPSS 18.0 software package (IBM, Armonk, NY, USA).

### Statistical Analysis

The data were expressed as the mean ± SD and were analyzed using SPSS statistical software (IBM, Armonk, NY, USA). Student's *t*-tests were used to compare pairs of means. The level of significance was set as *p* < 0.05. The datasets generated during and/or analyzed during the current study are available from the corresponding author on reasonable request.

## Results

### Screening Protein Spots With Potential Value

**Figure 1:**
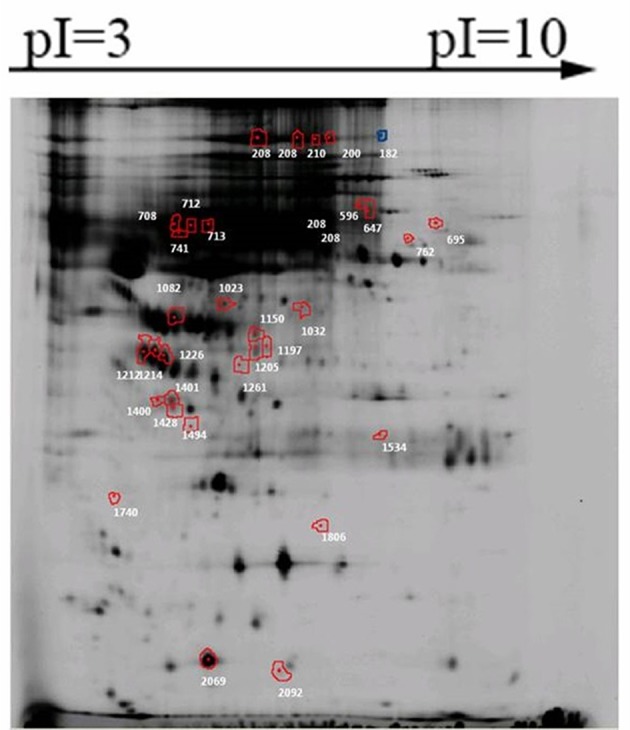
Significantly differently expressed protein spots between pSS patients with and without NMOSD. Proteins of each spot represented were listed in Table 2.

Firstly, the proteins that present in all gels from one group were selected. Secondly, the abundance of these protein spots was compared between pSS patients with and without NMOSD, and a total of 206 protein spots were found significantly differently expressed in the screening group. Thirdly, the relative abundance of proteins spots with potential value was estimated 1.5 times higher or lower than expressed in the other group. Thirty-two of the 206 proteins spots were found to be of potential value (Figure 1). Among them, the abundance of 16 protein spots was increased in pSS without NMOSD, while the other 16 protein spots decreased.

**Table 2:** Characteristics of differential protein spots from pSS with NMOSD, pSS without NMOSD.

**Spot No**.	**MW**	**pI**	**Protein name**	**Fold change (pSS with/without NMOSD)**
208	143534.4	6.32	complement factor H	2.28
596	77007.2	6.81	Serotransferrin	1.77
708	52384.6	6.55	Hemopexin	−2.57
712	52384.6	6.55	Hemopexin	−1.77
713	54789.8	5.56	Alpha-1B-glycoprotein	−1.76
741	52384.6	6.55	Hemopexin	−2.36
762	66288.9	7.22	macrophage-stimulating 1-like protein	−2.25
1023	39602.5	5.28	CD5 antigen	−2.08
1082	31647	8.48	HP protein	3.4
1150	31647	8.48	HP protein	3.15
1212	53031.2	5.89	Clusterin	−2.4
1214	62254.9	5.14	Keratin	−2.41
1401	30329.6	6.01	Alpha-1-microglobulin	−1.77
1428	30329.6	6.01	Alpha-1-microglobulin	−1.53
2902	45860.8	6.13	Haptoglobin	−3.61
206			N/A	2.4
1226			N/A	−2.26
1400			N/A	−1.65
1740			N/A	−1.58
1806			N/A	−1.58

*pSS, primary Sjögren's syndrome; NMOSD, neuromyelitis optica spectrum disorder; MW, molecular weight; pI, isoelectric point*.

### Identification of Protein Spots With Potential Value by MALDI-TOF/TOF MS

Only 20 out of the 32 protein spots were found in 2-DE gels, and 9 candidate proteins were identified: complement factor H (CFH), hemopexin, alpha-1B-glycoprotein, putative macrophage-stimulating 1-like protein, CD5 antigen-like OS, HP protein, clusterin, keratin (type I cytoskeletal 9 OS), alpha-1-microglobulin (Table 2). Based on a review of related literature, CFH and clusterin might be related to immune diseases, and thus they were chosen for further verification.

### Verification of Serum Clusterin and CFH

**Figure 2:**
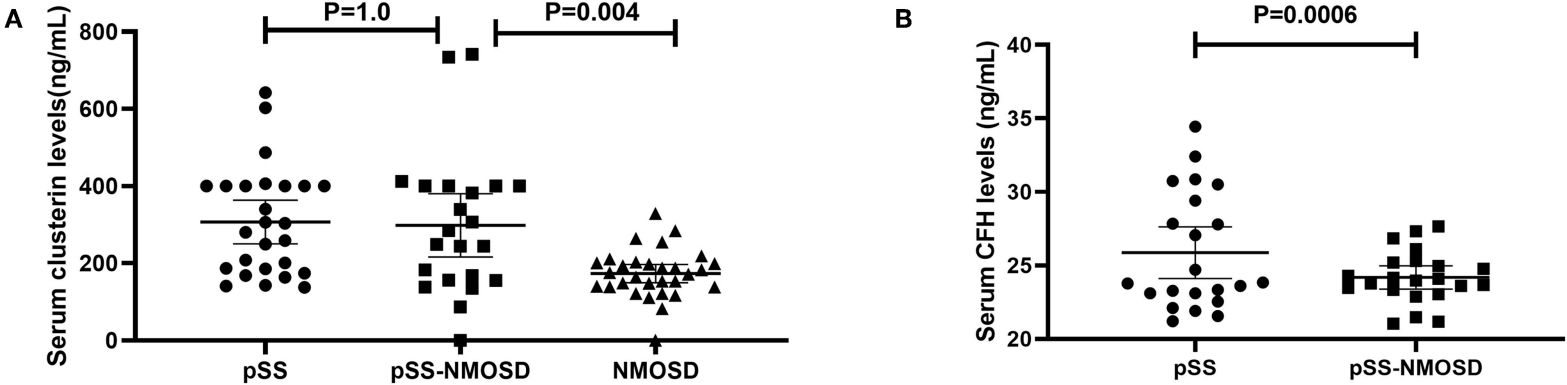
Verification of clusterin and CFH by ELISA asssys. **(A)** validation of serum clusterin levels; **(B)** validation of serum CFH levels.

The levels of clusterin and CFH in sera were quantified by ELISA to confirm the altered expression that was revealed by proteomic analysis and to validate their potentials as biomarkers of pSS with NMOSD. The results of DIGE were validated firstly. In the screening group, the level of clusterin was the same with DIGE while the level of CFH was not. Serum levels of clusterin and CFH were then determined in the verification group by ELISA. Results showed that serum clusterin was higher in pSS without NMOSD than with NMOSD but not significant (307.26 ± 140.26 vs. 298.33 ± 184.52 ng/ml, *p* = 1.00). However, serum clusterin was significantly higher in NMOSD with pSS than without (298.33 ± 184.52 vs. 173.49 ± 63.03 ng/ml, *p* < 0.01). On the other hand, the levels of CFH were lower in pSS patients with NMOSD than without NMOSD (24.19 ± 1.79 vs. 25.87 ± 3.98 ng/ml, *p* < 0.01). ELISA results for serum levels of clusterin and CFH in the verification group are shown in Figure 2.

In statistical analysis, the period that onset of disease to diagnosis was significantly different between SS with and without NMOSD in the screening group, while the age on disease onset was not. The SS with NMOSD, without NMOSD, and the NMOSD patients of the confirmation group were matched for most of the clinical condition, especially in the age of disease onset. We, therefore, considered that the differences we found come from disease heterogeneity.

## Discussion

In this research, DIGE combined with MALDI-TOF/TOF MS was applied to compare the protein pattern of serum from pSS patients with and without NMOSD. There were 9 proteins found to be significantly differently expressed between groups and could be potential biomarkers for pSS with NMOSD. The serum levels of alpha-1B-glycoprotein, alpha-1-microglobulin, CD5 antigen-like OS, clusterin, hemopexin, type I cytoskeletal 9, and putative macrophage-stimulating 1-like protein were lower in pSS patients with NMOSD than without NMOSD, while CFH and HP protein OS were higher in pSS patients with NMOSD. Based on literature review, clusterin and CFH, which may be relevant to related diseases, were further verified in this study.

Clusterin is a multifaceted protein functioning at the crossroads of inflammation and autoimmune diseases. The main form of clusterin is a secreted heterologous protein with a molecular weight of 80 kDa (21). In our study, serum clusterin was higher in pSS without NMOSD than with NMOSD but not significant. However, serum clusterin was significantly higher in NMOSD with pSS than without (Figure 2). Therefore, clusterin might be a potential biomarker that can differentiate NMOSD with and without pSS. Recent researches showed that clusterin could be found in saliva, tears, and salivary glands of pSS patients and participate in the pathogenesis of exocrine involvement (22–24), which suggest that increased clusterin might participate in the pathogenesis of NMOSD to complicated with pSS.

For the pathogenesis of NMOSD, binding of NMO-IgG to its target AQP4 initiates several immune processes. Often, the binding could readily activate the complement system, subsequently, amplify inflammation, and disrupt the blood-brain barrier, leading to astrocyte injury and causing secondary demyelination and neuronal loss (25). CFH is an important regulator in complement activation alternative pathways. It also acts as a co-factor for factor I to regulate the C3 and C5 convertases. Shi et al. (26) reported that engineered neural stem cells with CFH can attenuate inflammatory infiltration and immune-mediated damage of astrocytes. Moreover, Pohl et al. (27) reported that only T cells were seen in the lesions of destructive astrocyte from NMO patients. Of note, the T cells in the lesions showed signs of activation. Since T cell activation was regulated by the decreased expression of CFH in microglia, decreased CFH might be a risk factor for NMOSD. Interestingly, the levels of CFH were lower in pSS patients with NMOSD than without NMOSD (Figure 2), which is consistent with the assumption that decreased CFH is a risk factor for NMOSD.

There are three limitations in the current study. Firstly, observing the levels change of clusterin and CFH during treatment will support our finding to an extent. However, the rate of lost to follow-up is high that we could not perform this study. Secondly, comparing the levels change of selected proteins in cerebrospinal fluid among groups, which might act as an important confirmation of their pathogenesis role, were not done owing to ethical issues. Thirdly, the SS with NMOSD is a rare disease that limits the inclusion of related patients. The current sample size could help access a statically significant result, but not enough for generating a stable statistical result.

In conclusion, this is the first serum comparative proteomics focusing on the difference between pSS patients with and without NMOSD. Our results confirm that DIGE is a useful tool for such comparison. Increased clusterin and decreased CFH in pSS patients with NMOSD were potential biomarkers and related to the pathogenesis of these diseases. Further researches with larger sample size and more subgroup of patients are required to verify the value of these candidate biomarkers and their clinical significance.

## Data Availability Statement

The raw data supporting the conclusions of this manuscript will be made available by the authors, without undue reservation, to any qualified researcher.

## Ethics Statement

This study was carried out in accordance with the recommendations of the Ethics Committee of the Peking Union Medical College Hospital with written informed consent from all subjects. All subjects gave written informed consent in accordance with the Declaration of Helsinki. The protocol was approved by the the Ethics Committee of the Peking Union Medical College Hospital.

## Author Contributions

YL and YZ designed the research. LQ and CD performed the experiments. QW, WZ, YF, and YX recruited patients. CD wrote the manuscript and YL approved the version to be published.

### Conflict of Interest

The authors declare that the research was conducted in the absence of any commercial or financial relationships that could be construed as a potential conflict of interest.
